# Inquiry Concerning the Choice of Medical Books

**Published:** 1817-02

**Authors:** 


*For the London Medical and Physical Journal.*

*Inquiry concerning the Choice of Medical Books;* by A YOUNG BUT CONSTANT READER.

THE persuasion that you are not only willing but desirous of affording every assistance to the improvement of the junior members of the profession, emboldens me, at this time, to address you. I am apprenticed to a surgeon-apothecary and man-midwife, for the space of seven years. I have, and most likely shall continue to have, much time at my own command for reading, &c. My master gives me free access to his medical books, but will not trouble himself to direct my attention to those which would be most beneficial to me. Feeling I am very incompetent to judge for myself in this particular, shall esteem it a great favor if you would point out to me a course of reading in the different branches of medical science, (such as anatomy, practice of medicine, chemistry, botany, &c.) and the order in which the different books should be read. You are well aware, gentlemen, that I have to undergo two very rigorous examinations, when it will be necessary to have a good knowledge of these subjects, to enable me to pass the ordeal with credit to myself and satisfaction to my friends.

As it occurs to me, that keeping a common-place book, for the insertion of extracts and other particulars which may appear interesting, it would greatly enhance the obligation, if you would inform me of the most proper and suitable form for such a book, and of the best manner in which to keep it.

A full and explicit answer to the foregoing request in your next number, with any other advice you may think proper to give, will not only prove beneficial to myself, but to many others placed in similar circumstances.

*December* 16, 1816.

P. S.—I would beg leave to observe, that, in page 433 of your 34th volume, there are some remarks which bear upon my first request, but they do not enter into the subject so fully as I could wish.

